# Prevalence, Risk Factors, and Clinical Profiles of Hepatitis D Virus in Nigeria: A Systematic Review, 2009–2024

**DOI:** 10.3390/v16111723

**Published:** 2024-10-31

**Authors:** Victor Abiola Adepoju, Donald Chinazor Udah, Qorinah Estiningtyas Sakilah Adnani

**Affiliations:** 1 Department of HIV and Infectious Diseases, Jhpiego (an affiliate of Johns Hopkins University), Abuja 900918, Nigeria; 2 Laboratory for Toxicology and Micronutrient Metabolism, Department of Chemical Pathology, College of Medicine, University of Ibadan, Ibadan 200001, Nigeria; donald.udah@gmail.com; 3 JSI Research and Training Institute Inc. (JSI), Abuja 900108, Nigeria; 4 Department of Public Health, Universitas Padjadjaran, Bandung 40161, Indonesia; qorinah.adnani@unpad.ac.id

**Keywords:** Hepatitis D, viral hepatitis, prevalence, liver enzyme, risk factors, co-infection, superinfection, hepatocellular carcinoma, ELISA, phylogenetic analysis

## Abstract

Background: The World Health Organization (WHO) recommends hepatitis D virus (HDV) screening among hepatitis B virus (HBV) infected individuals, with a focus on priority populations in resource-limited settings like Nigeria. HDV infection is a growing public health challenge, particularly among individuals with chronic hepatitis B virus (HBV) infection. HDV accelerates liver disease progression and significantly increases the risk of cirrhosis and hepatocellular carcinoma. Despite this, the epidemiology of HDV in Nigeria remains inadequately documented. This scoping review critically evaluates the prevalence, risk factors, and clinical outcomes of HDV co-infection among HBV patients in Nigeria. Method: We conducted a systematic review following Preferred Reporting Items for Systematic Reviews and Meta-Analyses (PRISMA) 2020 guidelines. The review included observational cross-sectional studies published between 2009 and 2024. We focused on studies that used Immunoglobulin G (IgG) antibody testing or RNA-based diagnostics to assess HDV prevalence. We included PubMed, Google Scholar, and Dimensions databases due to their broad indexing and coverage of peer-reviewed articles and accessibility. We screened the studies for their relevance to HDV prevalence, risk factors, and clinical outcomes, while excluding those that only tested for IgM or HDV antigen. Eleven studies, with a combined sample size of 2308 participants, were included in the final analysis. We performed a narrative synthesis of the findings, considering geographic, gender, and age-based variations in HDV prevalence and clinical impact. Results: HDV prevalence among HBV-infected individuals in Nigeria ranged from 2.0% to 31.6%. The highest prevalence was reported in the Southwest (31.6%) among malaria patients, while lower rates were observed in the Southeast (2.8%). Prevalence was higher in males, particularly those aged 21–30 years in the Southwest and 31–40 years in other regions. RNA-based testing provided more accurate data on active viremia, with viremic HDV prevalence rates ranging from 3.2% to 16%. Triple infection with HIV/HBV/HDV was associated with significantly lower CD4+ cell counts and worse clinical outcomes, including elevated liver enzymes and rapid progression to liver cancer. Key risk factors for HDV co-infection included multiple sexual partners, sharing of needles, and unsafe medical practices. Co-infected patients demonstrated worse clinical outcomes, such as elevated liver enzymes, decompensated cirrhosis, and higher rates of hepatocellular carcinoma. Conclusions: Our review underscores the urgent need for routine HDV screening among HBV patients in Nigeria, especially given the severe clinical consequences of co-infection. The recent WHO guidelines recommending HDV screening align with our findings, which emphasize the importance of RNA-based HDV testing among HBV-positive patients to improve diagnostic accuracy. Public health efforts should prioritize tailored interventions based on geographic, age, and gender disparities in HDV prevalence. Triple infection with HIV/HBV/HDV requires integrated care models to address both immune suppressions as indicated by diminished CD4 cell count and liver disease progression, as these patients face worse outcomes. Targeted HDV screening in mostly affected demographics and geographies and improved Nigeria capacity for cheaper HDV RNA/PCR diagnostics can reduce liver-related morbidity and mortality caused by HBV, which can be worsened and accelerated by HDV coinfection.

## 1. Introduction

Hepatitis D virus poses a significant public health challenge globally, particularly among millions suffering from chronic HBV infection [1]. According to the World Health Organization, HDV-HBV coinfection may account for around 20% of the cases of liver disease and liver cancer among HBV-infected individuals [1]. The HDV is a satellite RNA virus that relies on HBV for replication. Discovered in 1977 by Mario Rizzetto, HDV is the smallest known virus infecting humans and utilizes host RNA polymerase for its lifecycle [2]. Approximately 4.5% of individuals positive for the hepatitis B surface antigen (HBsAg) are also infected with HDV. This translates to about 12 million HBV patients coinfected with HDV worldwide [3]. Transmission of HDV can occur concurrently with HBV (co-infection) or after HBV infection (superinfection).

The epidemiology of HDV in Nigeria is intricate and influenced by regional disparities and the interplay with co-infections such as HIV and Hepatitis C Virus (HCV). Studies indicate significant regional variations in HDV prevalence, with factors such as HBV birth dose vaccination coverage and the presence of other viral infections playing crucial roles [4,5]. For instance, in Abuja, Nigeria, the seroprevalence of HDV antibodies among chronic HBV patients was found to be 18.9%, with those positive for HDV showing more severe liver function test (LFT) abnormalities compared to those without HDV infection [6].

HDV infection exacerbates the progression of liver diseases associated with HBV, leading to rapid progression to cirrhosis and hepatocellular carcinoma (HCC) [7]. Risk factors for HDV in Nigeria include perinatal transmission from HBsAg-positive mothers and sexual contact with infected individuals. HDV infection significantly increases the risk of cirrhosis and HCC and complicates liver disease management, and this often necessitates liver transplantation [8,9,10].

Global studies highlight the severe impact of HDV on liver disease progression. For instance, in a North American cohort, HDV was significantly associated with intravenous drug use and elevated alanine aminotransferase levels, suggesting a need for targeted screening in high-risk populations [11]. In Mongolia, collaborative efforts in infection control have significantly reduced the incidence of acute HDV infections, which underscores the importance of preventive strategies [12]. In Nigeria, a community survey in Cross River State found a 5.6% prevalence of anti-HDV among asymptomatic HBsAg carriers, with no significant associations with assessed risk factors [9].

The introduction of HBV vaccination has positively impacted HDV infection rates, yet a substantial portion of HDV-positive individuals show no identifiable risk factors, indicating the need for broader screening measures [13]. In 2024, the World Health Organization (WHO) recommends universal HDV antibody testing for all individuals with chronic hepatitis B (CHB), or, if capacity is limited, prioritized testing for high-risk populations. According to the WHO, those who test positive for HDV antibodies should then undergo a nucleic acid test (NAT) to confirm active infection. WHO also recommends reflex testing, a situation where HDV antibody and RNA testing are conducted following a positive HBsAg result, to improve diagnosis and treatment [1]. Given the severe health outcomes associated with HDV infection and the economic challenges of managing liver disease in high-burden countries like Nigeria, there is an urgent need for comprehensive strategies to address these issues. The objective of the study is to systematically review the prevalence, risk factors, and clinical profiles of HDV in Nigeria. By synthesizing data from HDV studies conducted in Nigeria, this analysis aims to inform public health policies, enhance screening and vaccination programs, and tailor treatment strategies to address the unique challenges posed by HDV in the Nigerian context.

## 2. Materials and Methods

### 2.1. Study Design

This systematic review examines the prevalence, risk factors, and clinical profiles of Hepatitis D Virus (HDV) in Nigeria from 2009 to 2024. We followed the PRISMA 2020 guidelines for the review. However, no formal protocol was published due to time constraints and the urgent need for synthesized evidence in this evolving research area. The aim was to consolidate existing knowledge while identifying gaps for future inquiry.

### 2.2. Search Strategy

We conducted the literature search across PubMed, Google Scholar, and Dimensions databases. One author (VAA) performed the search between 1 May and 15 September 2024, using the following keywords: “Hepatitis D,” “Delta Hepatitis,” “Prevalence,” and “Nigeria.” We sought articles written in English and published from 2009 to 2024. Table 1 summarizes the search queries used in the databases.

### 2.3. Inclusion and Exclusion Criteria

We included observational studies (cross-sectional, cohort, and case-control) that reported HDV prevalence, risk factors, or clinical characteristics in human subjects in Nigeria. Only studies published in English with accessible full texts were considered. For accurate HDV prevalence, we included studies that utilized IgG antibody or HDV RNA testing. we excluded studies not focused on HDV, conducted outside Nigeria, or lacking primary outcomes. We also excluded studies testing only for HDV antigen or IgM antibodies, as well as non-observational studies (systematic reviews, editorials, letters, or case reports) and those with insufficient data or overlapping datasets. We included only observational studies since this systematic review is exploratory and aims to summarize the information on HDV prevalence, risk factors, and clinical characteristics in Nigeria. These studies are limited in addressing long-term impacts, but their inclusion broadens HDV prevalence and associated factors. Thus, synthesizing cross-sectional findings can provide a great overview of the existing evidence, paving the way for future longitudinal research. Currently, studies published in Nigeria on HDV are cross-sectional, and there is no published systematic review or meta-analysis on this subject.

### 2.4. Study Selection Process

**Figure 1:**
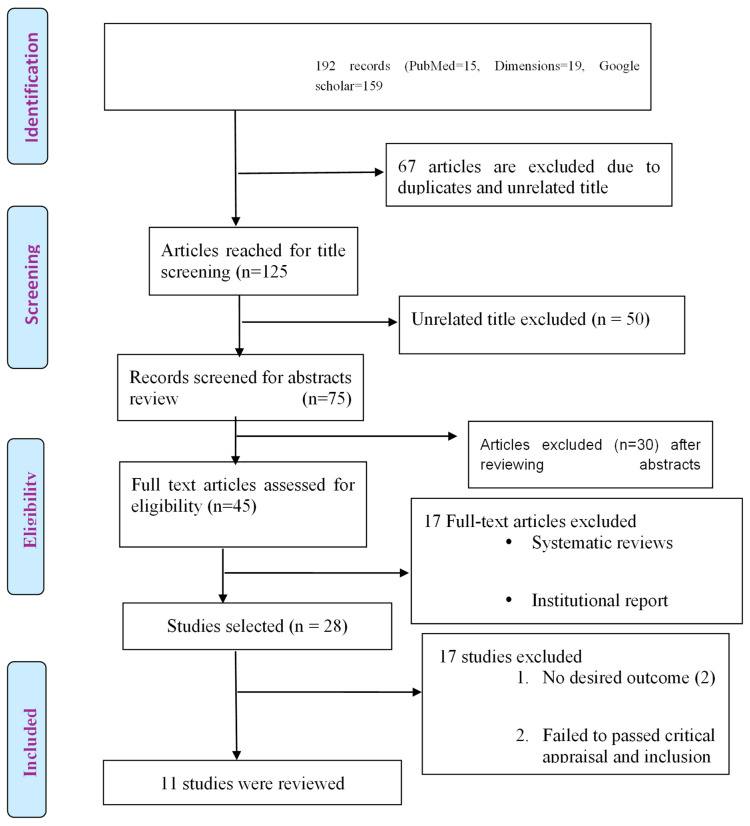
PRISMA Flowchart: Prevalence, risk factors, and clinical profiles of Hepatitis D in Nigeria: A systematic review.

After retrieving the articles, we screened titles and abstracts to remove duplicates and irrelevant studies. We conducted a search for relevant studies using predefined keywords in selected databases, applied a screening process, and removed duplicate records among the articles retrieved from multiple sources [14,15]. A full-text review of the remaining articles was conducted to assess alignment with the objectives of the review. Figure 1 below describes the flow of the literature selection process. The papers were further screened and evaluated based on their titles and abstracts. Any duplicate articles found to be the same in multiple databases were removed. The search results were generated based on terms in the specified databases, including Google Scholar (n = 159), PubMed (n = 15), and Dimensions (n = 19). The reviewers systematically identified potential biases and resolved discrepancies through consensus to ensure an unbiased evaluation and study selection. A total of 125 studies were initially identified. After a thorough review process, 28 studies were shortlisted. Of these, only 11 studies met the strict inclusion criteria of utilizing IgG antibody and/or RNA PCR testing for HDV, and these were included in the final analysis (Figure 1). Per the PRISMA 2020 recommendation, the list of all 28 included and excluded studies and reasons for their inclusion or exclusion are provided in the Supplementary Data (Table S1).

### 2.5. Data Extraction

The authors extracted relevant data using a standard Excel-based template. Two authors (VAA, DCU) independently extracted the data, and the results were reviewed and verified by a third author (QESA) for quality and clarity. The two authors independently assessed the full text of the potentially eligible publications. All disagreements were resolved by consensus. Initial agreement was achieved on 96% of the items, and discrepancies were discussed between authors until 100% agreement was ultimately achieved. Studies with an aggregate quality score of less than 13.5, or 50% of the maximum score, were regarded as poor quality. The following information was extracted from the included studies: author names and year of publication, type of HDV test, study location, study type, recruitment setting, study population, sample size, risk of bias, and summary of key findings (Table 2). Studies were classified as high, medium, or low risk of bias based on a quality assessment score.

### 2.6. Quality Assessment

We applied the Newcastle–Ottawa Scale (NOS) to assess the quality and risk of bias of the included studies [26]. This scale evaluates studies based on key domains: selection, comparability, and outcome assessment. Each study was scored based on the robustness of its methodology, sample size, and clarity in reporting outcomes. Studies that scored less than 50% of the maximum points were classified as having a high risk of bias and were excluded from the final analysis. Based on this evaluation, eleven studies were retained for inclusion.

### 2.7. Data Synthesis and Analysis

We performed a narrative synthesis of the key findings from the included studies. Given the heterogeneity in study populations and outcome measures, we did not perform a meta-analysis. Instead, we highlighted trends and variations in HDV prevalence across different populations and regions of Nigeria, as well as potential risk factors and clinical impact.

## 3. Results

### 3.1. Study Characteristics

This systematic review includes 11 cross-sectional studies that examine the prevalence, risk factors, and clinical impact of HDV co-infection among patients with chronic HBV infection in Nigeria. The studies were spread across a 12-year period, with the earliest in 2012 [22] and the most recent in 2023 [19]. The studies span across three Nigerian geopolitical zones and the Federal Capital Territory (FCT), while some were implemented in multiple states (National). Seven of the studies were conducted in the South (Southwest, Southeast), two from the North (Northwest, FCT), and two at the national level, i.e., multiple states [6,16,17,18,19,20,21,22,23,24,25]. The sample sizes range from 37 to 410 participants, and a total of 2308 participants were included in the study.

### 3.2. Antibody-Based HDV Prevalence

All 11 studies used ELISA to detect HDV antibodies. They all reported prevalence rates based on total anti-HDV (IgG and IgM) or IgG antibodies (see Table 3 below). These studies provide insight into both active and resolved HDV infections. The prevalence of HDV antibodies varied between 2.0% and 31.6% [6,16,17,18,19,20,21,22,24,25]. In the Southwest region, the prevalence ranged from 4.3% to 31.6%. A study from South-west Nigeria [16] reported the highest rate of 31.6% among presumptive malaria patients, while chronic HBV patients had lower rates of 4.3% [20] and 11% [17]. Among participants living with HIV, another study found an HDV antibody prevalence of 16% [18]. HDV prevalence was higher in the North. Among chronic HBV patients, a prevalence of 18.9% was reported in the FCT [6], and 10.8% was reported among HIV/HBV co-infected individuals in the Northwestern state of Sokoto [24]. In the Southeast, another study [25] reported a lower antibody prevalence of 2.8% among patients with chronic HBV/HCV co-infection. These findings demonstrate regional differences in HDV prevalence, with the highest rates found in the North and Southwest geopolitical zones. 

### 3.3. RNA-Based Viremic HDV Prevalence

Four studies used RNA-based testing to detect active HDV infection (see Table 4 below). These studies reported viremic rates that range from 3.2% to 16% [17,18,21,23]. For national studies that cut across multiple Nigerian states, a study [18] noted that 16% of HIV-positive participants who were positive for HBsAg had detectable HDV RNA. This study also highlighted the presence of HBV drug resistance mutations mainly associated with lamivudine resistance. This underscores the need for RNA-based testing to detect active HDV infections and manage drug-resistant strains. In the FCT, a study [21] found a 3.2% RNA-based prevalence of HDV among chronic HBV patients. HDV genotype 1 is the most prevalent in this study. In Southwest, another study [17] reported an 11% RNA-based prevalence among chronic HBV patients in Ogun State. These studies emphasize the importance of RNA-based testing for detecting ongoing viremia and differentiating between resolved and active infections.

### 3.4. Geopolitical Variation in Prevalence

**Figure 2:**
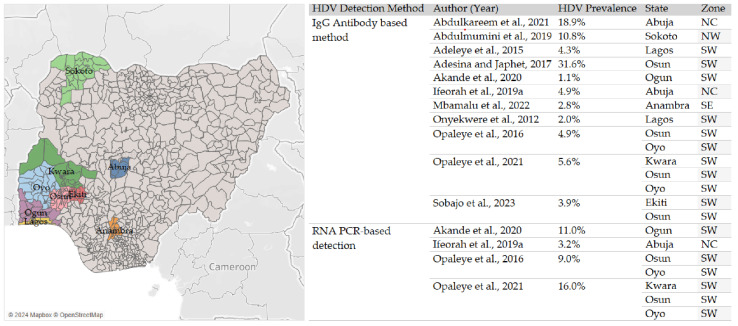
State distribution of HDV prevalence across Nigeria based on included studies, using antibody-based and RNA PCR-based diagnostic methods. Geographical regions: NC—North Central; NW—Northwest; SE—Southeast; SW—Southwest. The highest prevalence rates were reported in the Southwest and Northwest regions, with remarkable geographic disparities in HDV infection rates. References: [6,16,17,18,19,20,21,22,23,24,25].

The prevalence of HDV varied significantly across geopolitical zones in Nigeria. Four studies in the Southwest reported prevalence rates from 4.3% to 31.6%. The highest HDV rate was found among presumptive malaria patients [16]. RNA-based studies in this zone reported viremic rates of 11% and 16% [17,18]. In Sokoto (Northwest), [24] reported a prevalence of 10.8% among HIV/HBV co-infected individuals, slightly lower than in the Southwest but still significant. The FCT had the highest antibody-based prevalence of 18.9% [6], with a lower RNA-based prevalence of 3.2% [21]. The prevalence was lower in the Southeast, with an antibody prevalence of 2.8% [25]. These variations (higher burden observed in the North and Southwest zones) signal the influence of geographical factors on HDV prevalence. See Figure 2 and Table 5 below.

### 3.5. Age and Gender Distribution

Multiple studies explored the distribution of HDV prevalence across age and gender (see Table 6 below). Several studies reported higher HDV prevalence among males, especially those co-infected with HIV or chronic HBV [6,16]. For example, in the study by HBV/HDV co-infection among malaria patients [16], 83.3% of male patients were co-infected with HBV and HDV. Similarly, male predominance was seen in RNA-positive cases, where 64.7% of those with HDV RNA were male [23]. Another study [6] reported that IgG anti-HDV was positive in 13 (11.2%) of the males and 11 (17.2%) of the females. HDV prevalence also showed variations across age groups. Younger populations, especially those in the 21–30-year age group, had a higher prevalence in the Southwest, while in other regions, the 31–40-year age group had the highest prevalence [16,23].

### 3.6. Risk Factors for HDV Co-Infection

Four studies identified key risk factors for HDV co-infection (See Table 7 below). Multiple sexual partners, sharing of needles, past history of jaundice, and blood transfusions were the most reported risk factors [6,16,19,24]. In the FCT, patients with a history of jaundice were more likely to be co-infected with HBV/HDV [6]. In the national study by Sobajo et al. (2023) [19], individuals with multiple sexual partners and those who shared sharp objects were found to have a higher risk of HDV co-infection.

### 3.7. Clinical Implications of HDV Co-Infection

HDV co-infection worsened clinical outcomes in chronic HBV patients. Higher levels of liver enzymes Alanine transaminase (ALT) and Aspartate aminotransferase (AST) were reported among HDV-positive individuals in multiple studies (See Table 8 below). These indicate worse liver function [6,20]. HDV co-infection has been shown to exacerbate liver disease severity among CHB patients. A study [6] reported that 18.9% of CHB patients in Abuja were positive for HDV antibodies. These patients had significantly worse liver function tests and higher Child–Turcotte–Pugh (CTP) scores compared to HDV-negative patients. Specifically, mean serum levels of ALT, AST, albumin, and INR were significantly elevated in HDV-positive subjects (16.5 ± 13.8 IU/L, 26.3 ± 32.6 IU/L, 38.9 ± 7.6 g/L, and 1.2 ± 0.2, respectively) compared to HDV-negative subjects (10.8 ± 9.5 IU/L, 13.4 ± 11.2 IU/L, 41.4 ± 6.0 g/L, and 1.1 ± 0.2, respectively; *p* < 0.05). The mean CTP scores were 6.1 ± 2.1 for HDV-positive and 5.5 ± 1.2 for HDV-negative subjects (*p* = 0.03). Co-infection was also associated with more severe liver disease, including decompensated cirrhosis and hepatocellular carcinoma [25]. The study [25] observed that HBV viral load and alpha-fetoprotein (AFP) levels were higher in HBV/HDV coinfected patients compared to those with HBV mono-infection. Although the viral load differences were not statistically significant, the elevated AFP levels suggest a greater risk of liver cancer in these patients. Among HIV-positive patients, HDV co-infection was associated with more advanced liver disease and poorer clinical outcomes [18].

In summary, the 11 studies reviewed show a wide range of HDV prevalence, from 2.0% to 31.6%. Higher rates were observed and reported in the North and Southwest reions. RNA-based testing provided more accurate estimates of active viremia compared to antibody-based methods. Co-infection with HDV significantly worsened clinical outcomes, stressing the need for routine HDV screening and comprehensive management strategies for patients with chronic HBV in Nigeria.

## 4. Discussion

### 4.1. HDV Prevalence and Geographic Disparities in Nigeria

HDV prevalence in Nigeria shows significant regional variation, with rates ranging from 4.3% to 31.6% in the Southwest [16,17], 18.9% in the FCT [6], and 10.8% in the Northwestern state of Sokoto [24]. However, studies from North–Central state of Benue and the Northwestern state of Kano reported HDV antigenic prevalence of 2.7% and 5.43%, respectively, among HBsAg-positive individuals [27,28]. This could mean that healthcare access, socioeconomic factors, and cultural practices affect HDV disease transmission. However, two studies conducted in Cross rivers and Ekiti state Nigeria found zero HDV cases among patients on ART and pregnant women [29,30]. Another study in Nigeria [5] similarly noted regional differences in HBV prevalence. This further reinforces the idea that HDV hotspots overlap with HBV-endemic areas, as HDV occurs only in HBV-infected individuals. It is crucial to regionalize public health strategies for HDV, particularly in regions like the Northeast, where cross-border transmission from Cameroon may contribute to higher HDV rates [31]. Notwithstanding, reports that global estimates of HDV are likely underreported, particularly in low-resource settings like Nigeria [3,7], further echo the importance of localized data (as reported in this study), to fully understand the buren of HDV. Routine HDV screening and interventions such as HBV birth dose vaccination tailored to regional needs could, therefore, mitigate the impact of the virus in high-risk areas.

### 4.2. Impact of Demographics (Age and Gender) on HDV Prevalence

Across the 11 studies, it was observed that males had a higher prevalence of HDV co-infection, particularly in studies like [21], which reported that 64.7% of individuals with HDV RNA were male. This male predominance is consistent with findings from [32] (HDV was 13% among males and 10.5% among females) and that of [9,33], which further noted that males had higher liver enzyme levels and were more likely to experience HBV/HDV co-infection-related complications. Two additional studies also reported high co-infection rate in male compared to female [34,35]. In contrast, a study from Plateau state, Nigeria [36] found a higher prevalence of HDV among females (14.8%) vs. 5.5% in males, particularly those co-infected with HIV. This suggests that risk behaviors associated with gender may vary based on regional and population characteristics. The variation in diagnostic methods, with this study [36] using IgM detection, may also reflect differences in acute vs. chronic infection stages, which complicates direct comparisons with studies that used RNA PCR or IgG to assess chronic HDV prevalence.

The influence of age was also obvious from the review. Two studies [16,23] reported the highest HDV in younger to middle-aged populations, particularly those aged 21–30 and 31–40 years, respectively, in Southwest Nigeria. Also, HBV/HDV co-infection was highest among those less than 20 years in Abuja [35]. A study reported that HDV prevalence peaked among individuals aged 41–50 years [37], especially those with multiple sexual partners, while another study [6] found no significant association between age and HDV infection. The authors further noted that the mean age of anti-HDV+ (37.1 years) and anti-HDV- (34.8 years) participants was not statistically different. This variation may reflect differences in exposure to risk factors across age cohorts, such as changes in healthcare practices or generational shifts in disease awareness and prevention. The demographic variations in HDV prevalence suggest that public health interventions must be tailored to address both age-specific and gender-specific risk factors [5].

### 4.3. Diagnostic Methods and Their Impact on Prevalence Estimates

The reviewed studies predominantly used ELISA to detect anti-HDV antibodies. Only four studies used RNA-based testing to detect active HDV viremia in addition to antibody testing [17,18,21,23]. The RNA-based studies reported viremic rates between 3.2% and 16%, which underscores the importance of differentiating between resolved and active infections. A systematic review [38] earlier noted that RNA testing is the gold standard for identifying active HDV infection, and relying solely on antibody-based methods can lead to an underestimation of the true burden. In Nigeria, where healthcare infrastructure is limited, RNA testing is expensive and not widely available, leading to challenges in diagnosing and managing HDV. Moreover, [39] pointed out that variability in diagnostic methods across studies complicates comparisons of HDV prevalence. For instance, antibody-based methods may detect individuals who have cleared the infection but retain antibodies, while RNA-based tests provide a more accurate assessment of ongoing viral replication. This is crucial for understanding the transmission dynamics of HDV and for informing treatment strategies.

### 4.4. Risk Factors: Multiple Sexual Partners, Co-Infections, and Unsafe Medical Practices

Multiple sexual partners, sharing of sharp needles, and unsafe medical practices such as unregulated/unsafe blood transfusions were recurrent risk factors for HDV across the studies. A study [19] demonstrated that individuals engaging in high-risk sexual behaviors, such as having multiple sexual partners and sharing needles, were significantly more likely to be co-infected with HDV, as these are overlapping risk factors for both HBV and HDV infections. Other risk factors cited in the literature that increased HDV co-infection include unsafe blood transfusion and unsterilized surgical equipment [27,33,40]; sociocultural practices such as female circumcision and injection by non-certified healthcare practitioners [9]; chronic liver disease [20]; and history of jaundice [6]. Additional observations reflect global findings from a study by [41], who found that injection drug users were disproportionately affected by HDV. However, injection drug use as a driver of HDV is less commonly reported in Nigeria compared to other regions. Conversely, in Nigeria, traditional medical practices and sociocultural behaviors play a more prominent role. For instance, practices such as female circumcision and tribal rituals involving bloodletting or scarification often involve the use of non-sterilized instruments or the reuse of needles in informal healthcare settings, significantly contributing to the spread of HDV, particularly in rural areas where healthcare regulation is weaker. This aligns with Hayashi et al., 2021 [42], who emphasized the need for improved screening and infection control measures in healthcare settings to prevent HDV transmission. Addressing these practices requires culturally sensitive health education that respects local customs while promoting safe alternatives to mitigate the spread of HDV. For instance, health education programs could focus on the risks associated with non-sterilized instruments and encourage communities to adopt safer practices or seek medical procedures from certified practitioners. The global response to these risk factors has varied, reflecting the diverse contexts in which HDV transmission occurs. In high-income countries, the focus is often on harm reduction strategies, such as needle exchange programs and safe sex promotion [43]. These strategies are tailored to regions where intravenous drug use is a significant transmission route. In contrast, in low-resource settings like Nigeria, public health strategies must prioritize improving healthcare infrastructure, ensuring safe medical practices, and enhancing public awareness about the risks associated with certain traditional practices that promote HBV and HDV transmission. For example, implementing universal HBV vaccination programs can reduce the pool of HBsAg carriers, thereby indirectly lowering the risk of HDV transmission.

### 4.5. Clinical Impact: Liver Disease Progression and Complications

The clinical impact of HDV co-infection on liver disease progression is profound. Available pieces of evidence indicate that HDV significantly accelerates liver damage compared to HBV mono-infection. Multiple studies [25] report elevated liver enzyme levels in HDV-co-infected patients, which is a pointer to a more severe liver injury. Similarly, another study [44] found higher liver enzyme levels (ALT and AST) in HDV-HBV co-infected patients compared to HBV mono-infected patients, although no significant difference was observed for ALP levels. The accelerated deterioration seen in HDV-co-infected individuals places them at higher risk for advanced liver diseases such as decompensated cirrhosis and hepatocellular carcinoma. Consistent with global literature, such as the findings of Oghlikhan et al, 2016 [4], these results underscore the aggressive nature of HDV in exacerbating liver pathology. This necessitates early diagnosis and management to mitigate long-term complications. In addition to elevated liver enzymes, other studies from Nigeria further highlight the clinical markers that indicate more rapid liver disease progression in HDV/HBV co-infected patients. Furthermore, another study [6] observed higher Child–Turcotte–Pugh scores, while in South-western Nigeria, a study [45] reported elevated β2-microglobulin levels beyond the normal level in individuals coinfected with HBV and HDV as compared with the mono-infected individuals. Both markers are associated with advanced liver disease and a greater likelihood of progression to cirrhosis and HCC. These biomarkers underscore the need for robust clinical monitoring and early intervention strategies to prevent irreversible liver damage in co-infected individuals.

Furthermore, the compounded risk in patients triple-infected with HDV, HBV, and HIV is evident. Baeka et al., 2022 [46] reported significantly lower CD4+ cell counts in triple-infected patients, suggesting a more profound impact on immune restoration compared to HIV/HBV co-infection alone. This worsened immune status increases susceptibility to opportunistic infections and leads to poorer clinical outcomes and higher alkaline phosphatase (ALP) levels, further reflecting the heightened liver dysfunction caused by triple infections. These findings echo the need for integrated care models that address the complex clinical management of HDV/HBV co-infected patients, particularly those with concurrent HIV infection. Early detection and the integration of tailored antiviral treatments are critical to slowing disease progression and improving patient outcomes. For Nigeria, where access to advanced HDV diagnostics and treatments may be limited and costly, policy implications include expanding access to HDV testing infrastructure, particularly in high-risk populations, and strengthening the capacity of the national health system to manage HIV/HBV/HDV triple infections and HBV/HDV co-infections, which will be crucial to reducing liver-related morbidity and mortality in this population. Prioritizing early intervention and comprehensive HBV/HDV care models, Nigeria’s healthcare systems can mitigate the rapid progression of liver disease in patients with HDV co-infection. This will ultimately prevent severe outcomes such as cirrhosis and HCC and improve survival rates among vulnerable populations.

## 5. Conclusions

In summary, the reviewed studies reveal a complex interplay between diagnostic capacity, regional epidemiology, and clinical outcomes of HDV in Nigeria. The inclusion of RNA-based testing in routine HDV screening protocols, combined with public health interventions targeting high-risk populations, could significantly reduce the burden of HDV in Nigeria. The male predominance in HDV infections, particularly among those with higher-risk behaviors such as multiple sexual partners or sharing of needles, points to the need for targeted interventions. The clinical impact of HDV co-infection, particularly its role in accelerating liver disease progression, reflects the urgent need for comprehensive HDV screening and management programs in Nigeria. Geographic disparities in HDV prevalence further pinpoint the need for region-specific public health strategies that address the unique challenges of each zone/region. Furthermore, effective HDV drugs have remained elusive for decades. Antiviral therapy for HDV in Nigeria primarily consists of pegylated interferon-alpha and tenofovir, which are known to be associated with post-treatment relapses. Although the novel anti-HDV drug bulevirtide is not currently a widely accessible treatment option in Nigeria, its mention in broader global discussions points to the ongoing need for research and development of affordable HDV treatments for resource-limited settings like Nigeria. Ultimately, addressing the burden of HDV in Nigeria will require coordinated efforts to improve HDV diagnostics, enhance public health education, and raise awareness of the peril of HBV/HDV co-infection, including chronic liver disease, among younger populations in Nigeria. In addition, studies highlight the significant immune suppression observed in HIV/HBV/HDV co-infected individuals, particularly through reduced CD4+ cell counts, which exacerbates their vulnerability to opportunistic infections. This underscores the critical need for integrated care models that address not only liver disease progression but also immune restoration in co-infected populations. Moving forward, a coordinated approach that integrates improved diagnostics, comprehensive treatment strategies, and region-specific public health policies is essential to mitigate the impact of HDV and ensure better health outcomes for high-risk populations across Nigeria.

## Figures and Tables

**Table 1:** Search Strategy.

S/N	Database	Query	Results
1	PubMed	“Hepatitis D” OR “HDV” OR “Delta Hepatitis” AND “Nigeria”	15
2	Google Scholar	In title: “Hepatitis D” OR “HDV” OR “Delta Hepatitis” AND “Nigeria”	164
3	Dimensions	“Hepatitis D” OR “HDV” OR “Delta Hepatitis” AND “Prevalence” AND “Nigeria”	19

**Table 2:** Key characteristics of the included study.

S/N	Author (Year)	Sample Size	Study Region	Population	Setting	Risk of Bias
1	Abdulkareem et al., 2021 [6]	180	FCT (North)	Chronic HBV patients	Hospital	Medium
1	Adesina and Japhet, 2017 [16]	275	Southwest (Osun)	Malaria patients	Hospital	Medium
2	Akande et al., 2020 [17]	99	Southwest (Ogun)	Chronic HBV patients	Hospital	Medium
4	Opaleye et al., 2021 [18]	310	National (Osun, Oyo and Kwara)	HIV-positive patients	Hospital	Medium
5	Sobajo et al., 2023 [19]	410	National (Osun and Ekiti)	General Population	Hospital	Medium
6	Adeleye et al., 2015 [20]	186	Southwest (Lagos)	Chronic HBV patients	Hospital	Medium
7	Ifeorah et al., 2019a [21]	306	FCT (North)	HBV/HIV coinfected	Facility	Low
8	Onyekwere et al., 2012 [22]	245	Southwest (Lagos)	Chronic HBV patients	Facility	Medium
9	Opaleye et al., 2016 [23]	188	Southwest (Oyo and Osun)	Chronic HBV patients	Hospital	Medium
10	Abdulmumini et al., 2019 [24]	37	Northwest (Sokoto)	HIV/HBV coinfected	Facility	Medium
11	Chinneye et al., 2022 [25]	72	Southeast (Anambra)	Chronic HBV/HCV patients	Facility	Medium

**Table 3:** Antibody-based HDV prevalence.

S/N	Author (Year)	Test Type (Antibody)	Antibody Type (If ELISA)	Manufacturer	Antibody Prevalence (IgG/Total)
1	Abdulkareem et al., 2021 [6]	ELISA	Total (IgG and IgM)	Creative Diagnostics	18.9% HDV antibodies
2	Adesina and Japhet, 2017 [16]	ELISA	Total (IgG and IgM)	Diapro Italy	31.6% HDV
3	Akande et al., 2020 [17]	ELISA	Total (IgG and IgM)	Diapro Diagnostic	1.1% HDV antibodies
4	Opaleye et al., 2021 [18]	ELISA	Total (IgG and IgM)	Wantai Diagnostics	5.6% antibodies
5	Sobajo et al., 2023 [19]	ELISA	Total (IgG and IgM)	Not Stated	3.9% IgG, 1.9% IgM
6	Adeleye et al., 2015 [20]	ELISA	IgG	Not Stated	4.3% antibodies
7	Ifeorah et al., 2019a [21]	ELISA	Total anti-HDV	DiaSorin, QIAGEN	4.9% antibodies
8	Onyekwere et al., 2012 [22]	ELISA	IgG	Diapro Diagnostic	2.0% antibodies
9	Opaleye et al., 2016 [23]	ELISA	Total (IgG and IgM)	DiaSorin, QIAGEN	4.9% antibodies
10	Abdulmumini et al., 2019 [24]	ELISA	IgG	Perfemed USA	10.8% antibodies
11	Chinneye et al., 2022 [25]	ELISA	IgG	Melsin Medical	2.8% antibodies

**Table 4:** RNA-Based Viremic HDV Prevalence.

S/N	Author (Year)	Test Type (RNA)	Manufacturer	Prevalence of RNA	Key Findings
1	Akande et al., 2020 [17]	RNA Testing	Diapro Diagnostic	11% HDV RNA	Detected in chronic HBV patients
2	Opaleye et al., 2021 [18]	RNA Testing	Wantai Diagnostics	16% RNA positive	Highlighted HBV drug resistance
3	Ifeorah et al., 2019a [21]	RNA Testing	DiaSorin, QIAGEN	3.2% RNA positive	Detected mainly HDV Genotype 1
4	Opaleye et al., 2016 [23]	RNA Testing	DiaSorin, QIAGEN	9% RNA positive	Found in Oyo and Osun states

**Table 5:** Geopolitical Variation in HDV Prevalence.

S/N	Author (Year)	Region	Prevalence Range	Highest Reported Prevalence
1	Abdulkareem et al., 2021 [6]	FCT	18.90%	18.9% antibodies in FCT
2	Adesina and Japhet, 2017 [16]	Southwest	31.6%	31.6% in Osun (malaria patients)
3	Akande et al., 2020 [17]	Southwest	11%	11% RNA-based in Ogun
4	Opaleye et al., 2021 [18]	National	16% RNA-based	16% RNA in multistate study
5	Abdulmumini et al., 2019 [24]	Northwest	10.80%	10.8% in Sokoto
6	Chinneye et al., 2022 [25]	Southeast	2.80%	2.8% in Anambra

**Table 6:** Age and gender distribution in HDV prevalence.

S/N	Author (Year)	Prevalence in Males	Prevalence in Females	Age Group with Highest Prevalence
1	Abdulkareem et al., 2021 [6]	11.2% males	17.2% females	
2	Adesina and Japhet, 2017 [16]	83.3% male co-infection	16.7% females	21–30 years
3	Opaleye et al., 2016 [23]	64.7% males	35.3% females	31–40 years

**Table 7:** Risk Factors for HDV Co-Infection.

S/N	Author (Year)	Key Risk Factors Identified	Region	Study Population
1	Abdulkareem et al., 2021 [6]	History of jaundice, multiple sexual partners	FCT	Chronic HBV patients
2	Adesina and Japhet, 2017 [16]	Multiple sexual partners, needle sharing	Southwest	Malaria patients
3	Sobajo et al., 2023 [19]	Needle sharing, blood transfusions	National	General Population
4	Abdulmumini et al., 2019 [24]	Unsafe medical practices	Northwest	HIV/HBV coinfected patients

**Table 8:** Clinical Impact of HDV coinfection.

S/N	Author (Year)	Clinical Impact	Prevalence of Severe Outcomes	Region
1	Abdulkareem et al., 2021 [6]	Worsened liver function, elevated liver enzymes	18.9% with severe liver damage	FCT
2	Adeleye et al., 2015 [20]	Higher liver enzymes, cirrhosis	4.3% co-infection with worse outcomes	Southwest
3	Chinenye et al., 2022 [25]	Advanced liver disease, cirrhosis	2.8% with advanced liver disease	Southeast

## Data Availability

The original contributions presented in the study are included in the article/Supplementary Materials, further inquiries can be directed to the corresponding author.

## Supplementary Materials

The following supporting information can be downloaded at: https://www.mdpi.com/article/10.3390/v16111723/s1. Table S1: List of Excluded Studies and Reasons for Exclusion Based on Diagnostic Methodology and PRISMA Guidelines, 2009–2024. References: [6,8,9,16,17,18,19,20,21,22,23,24,25,27,28,29,30,31,32,33,34,35,36,37,40,44,45,46].
