# Supplementary material for: Prevalence, Risk Factors, and Clinical Profiles of Hepatitis D Virus in Nigeria: A Systematic Review, 2009–2024
Source: Viruses. 2024 Oct 31;16(11):1723. doi: 10.3390/v16111723 (PMC11598852; doi:10.3390/v16111723)
Supplement: Supplementary file 1 [file viruses-16-01723-s001.zip › viruses-3238076-supplementary.pdf]

Table S1. List of Excluded Studies and Reasons for Exclusion Based on Diagnostic Methodology and PRISMA Guidelines, 2009–2024.

S/N	Author Name and Year of Publication	Type of HDV test (antigen, antibody, HDV RNA, multiple)	If ELISA, Type of Antibody Tested	Manufacturer Name	Location/State	Study Type	Recruitment Setting	Study Population	Sample Size	Risk of Bias	Summary of Key Findings	Included/excluded	Reason for Exclusion/inclusion
1	Okonkwo et al., 2022 [9]	ELISA	anti-HDV IgM antibody	Diagnostic Automation Inc, CA	Cross River	Cross-sectional	Community	Chronic HBV patients	90	Medium	5.6% prevalence of anti-HDV IgM; higher prevalence in females and larger families.	Excluded	IgM-only detection, not suitable for determining chronic HDV prevalence
2	Anejo-Okopi et al., 2021 [36]	ELISA	IgM	Mybiosource Inc, USA	Plateau state	Cross-sectional	Hospital	Chronic HBV patients	90	Medium	11.1% HDV infection; higher in females and HBV/HIV co-infected.	Excluded	IgM-only detection, not suitable for chronic prevalence
3	Okoror et al., 2017 [45]	ELISA	Not stated	WKEAMedical Supplies, China	Southwest Nigeria	Cross-sectional	Community-based	HBV and HDV mono- and co-infected individuals	368	Medium	17.9% HBsAg positivity; elevated serum β2-microglobul	Excluded	Type of ELISA antibody not stated

											in in coinfected individuals.		
4	Udosen et al., 2023 [40]	ELISA	Not stated	Bioassay, US	Cross River	Cross-sectional	Hospital	Preoperative patients	180	Medium	7.8% HBV, 1.1% HDV, 1.7% HCV prevalence	Excluded	Type of ELISA antibody not stated
5	Inyang-Etoh et al., 2018 [29]	ELISA	IgG	Mybiosource, Inc, San Diego, CA	Cross River	Cross-sectional	Hospital	HIV patients on HAART	250	Medium	8% HBV in HAART group; no HDV cases	Excluded	No HDV cases detected in study population
6	Nwika et al., 2023 [33]	ELISA	Not stated	Elabsciences	Rivers	Cross-sectional	Hospital	Chronic HBV patients	300	Medium	28.7% HBV, 10.4% HDV among HBV-positive donors	Excluded	Type of ELISA antibody not stated
7	Ajayi et al., 2021[44]	ELISA	IgM	ELISA Diagnostic Automation/Cortez Diagnostic Inc, Calabasas, USA	Borno	Cross-sectional	Hospital	Chronic HBV patients	180	Medium	3.3% HDV seroprevalence; higher liver enzyme levels in HDV-HBV co-infected patients	Excluded	IgM-only detection, not suitable for chronic prevalence
8	Nwokediuko & Ijeoma,	ELISA	Not stated	Diagnostic Automation Inc, CA	Enugu	Cross-sectional	Hospital	HBV-related liver disease	96	Medium	12.5% anti-HDV prevalence; higher in	Excluded	Type of ELISA antibody not stated

	2009 [32]							patients			advanced liver disease stages		
9	Ifeorah et al., 2017 [35]	ELISA	Not stated	Not stated	Abuja	Cross-sectional	Hospital	PLHIV on HAART	1102	Medium	10.3% HBV, 7.1% HBV/HDV, 0.7% HIV/HBV/HDV seroprevalence	Excluded	Type of ELISA antibody not stated
10	Ifeorah et al., 2019b [37]	ELISA	Not stated	Not stated	Abuja	Cross-sectional	Hospital	HBV-positive prospective blood donors	193	Low	HDV prevalence peaked at 41-50 years; 5.7% overall anti-HDV/HBV prevalence	Excluded	Type of ELISA antibody not stated
11	Okpokam, 2015 [34]	ELISA	IgM	Genway Biotech Inc, San Diego, USA	Cross River	Cross-sectional	Facility	CLD (76)+health subjects(15)	91	Medium	83.5% HBsAg positivity; 60.5% HBV/HDV co-infection	Excluded	IgM-only detection, not suitable for chronic prevalence
12	Waiya et al., 2018 [28]	ELISA	HDV Antigen	Not stated	Kano	Cross-sectional	Facility	Multiple Liver Diseases	115	Medium	5.43% HDV prevalence	Excluded	Antigen-only detection, not suitable for prevalence estimation

13	Mbaawuaga et al., 2014 [27]	ELISA	Not stated	Not stated	Benue	Cross-sectional	Mixed (facility and community)	Mixed (Pregnant women, blood donors, HCWs, sex workers)	1535	Medium	2.7% HDV antigen detection in HBsAg-positive individuals	Excluded	Antigen-only detection, not suitable for prevalence estimation
14	Chindah et al., 2024 [8]	ELISA	Not stated	PRO Diagnostic Bioprobes Srl, Italy	Plateau state	Cross-sectional	Facility	Children (1-16 years old)	180	Medium	4.4% HBsAg positivity; no HDV co-infection	Excluded	No HDV cases detected in study population
15	Baeka et al., 2022 [46]	ELISA	Not stated	Dia. Pro ELISA kit (Italy), SWE-Care rapid kit (China)	Rivers State	Cross-sectional	Hospital	HIV patients on HAART	93	Moderate	6.4% HDV prevalence among HIV patients; significant liver damage	Excluded	Type of ELISA antibody not stated
16	Egbebi et al., 2022 [30]	ELISA and rapid kit	Not stated	Diaspot Diagnostics	Ekiti State	Cross-sectional	Pregnant women attending antenatal clinic	Pregnant women (18-44)	350	Moderate	No HDV detected among HBV-positive pregnant women	Excluded	Type of ELISA antibody not stated
17	Ifeorah et al., 2024 [31]	Multiple (ELISA, RNA)	Anti-HDV antibody	DiaSorin (ELISA), Eurobio HDV RNA kit	Multistate	Cross-sectional	Medical outpatient clinics	Asymptomatic chronic HBV carriers	1281	Low	4.8% anti-HDV prevalence; 19.7% active HDV RNA detection,	Excluded	Only anti-HDV antibodies mentioned, no clarity on type of ELISA used

											regional disparities noted		
18	Adesina and Japhet, 2017 [16]	ELISA	Total (IgG and IgM)	Diapro Italy	Osun	Southwest	Hospital	Malaria patients	275	Medium	13.8% HBsAg positivity; 31.6% HBV/HDV co-infection	Included	Study utilized IgG antibody and or HDV RNA testing for HDV diagnosis
19	Akande et al., 2020 [17]	Multiple (Antigen, RNA, ELISA)	Total (IgG and IgM)	Diapro Diagnostic	Ogun	Southwest	Hospital	Chronic HBV	99	Medium	11% HDV-Ag positivity; 1.1% anti-HDV antibodies	Included	Study utilized IgG antibody and or HDV RNA testing for HDV diagnosis
20	Abdulkareem et al., 2021 [6]	ELISA	Total (IgG and IgM)	Creative Diagnostics	Abuja	FCT	Hospital	Chronic HBV	180	Medium	18.9% HDV antibodies; worse liver function in HDV patients	Included	Study utilized IgG antibody and or HDV RNA testing for HDV diagnosis
21	Opaleye et al., 2021 [18]	Multiple (Antibody, RNA)	Total (IgG and IgM)	Wantai Diagnostics	Multistate	National	Hospital	HIV-positive	310	Medium	16% HBsAg positivity; 16% had detectable HDV RNA	Included	Study utilized IgG antibody and or HDV RNA testing for HDV diagnosis
22	Sobajo et al., 2023 [19]	Multiple (Antigen,	Total (IgG and IgM)	Not stated	Multistate	National	Hospital	General Population	410	Medium	3.9% IgG anti-HDV; 1.9% IgM anti-HDV	Included	Study utilized IgG antibody and or HDV RNA testing for

		ELISA)											HDV diagnosis
23	Adeleye et al., 2015 [20]	ELISA	IgG	Not stated	Lagos	Southwest	Hospital	Chronic HBV	186	Medium	4.3% anti-HDV prevalence	Included	Study utilized IgG antibody and or HDV RNA testing for HDV diagnosis
24	Ifeorah et al., 2019a [21]	Multiple (Antibody, RNA)	Total anti-HDV	DiaSorin, QIAGEN	Abuja	FCT	Facility	HBV/HIV coinfected	306	Low	4.9% HDV antibodies; 3.2% had HDV RNA	Included	Study utilized IgG antibody and or HDV RNA testing for HDV diagnosis
25	Onyekwere et al., 2012 [22]	ELISA	IgG	Diapro Diagnostic	Lagos	Southwest	Facility	Chronic HBV	245	Medium	2.0% tested positive for anti-HDV	Included	Study utilized IgG antibody and or HDV RNA testing for HDV diagnosis
26	Opaleye et al., 2016 [23]	Multiple (Antibody, RNA)	Total (IgG and IgM)	DiaSorin, QIAGEN	Oyo, Osun	Southwest	Hospital	Chronic HBV	188	Medium	9% had detectable HDV RNA; 4.9% anti-HDV antibodies	Included	Study utilized IgG antibody and or HDV RNA testing for HDV diagnosis
27	Abdulmumini et al., 2019 [24]	ELISA	IgG	Perfemed USA	Sokoto	Northwest	Facility	HIV/HBV	37	Medium	10.8% HDV among HIV/HBV coinfected	Included	Study utilized IgG antibody and or HDV RNA testing for HDV diagnosis

28	Chinneye et al., 2022 [25]	ELISA	IgG	Melsin Medical	Anambra	Southea st	Facility	Chronic HBV/HCV	72	Mediu m	2.8% HBV/HDV co-infection	Include d	Study utilized IgG antibody and or HDV RNA testing for HDV diagnosis
